# Isolation and differential transcriptome of vascular smooth muscle cells and mid-capillary pericytes from the rat brain

**DOI:** 10.1038/s41598-018-30739-5

**Published:** 2018-08-16

**Authors:** Stéphanie Chasseigneaux, Yasmine Moraca, Véronique Cochois-Guégan, Anne-Cécile Boulay, Alice Gilbert, Stéphane Le Crom, Corinne Blugeon, Cyril Firmo, Salvatore Cisternino, Jean-Louis Laplanche, Emmanuel Curis, Xavier Declèves, Bruno Saubaméa

**Affiliations:** 1 Inserm, U1144, Paris, F-75006 France; 2 Université Paris Descartes, UMR-S 1144, Paris, F-75006 France; 3 Université Paris Diderot, UMR-S 1144, Paris, F-75013 France; 4 IBENS, Département de Biologie, École normale supérieure, CNRS, Inserm, PSL Research University, F-75005 Paris, France; 5 Sorbonne Universités, UPMC Univ Paris 06, Univ Antilles, Univ Nice Sophia Antipolis, CNRS, Evolution Paris Seine - Institut de Biologie Paris Seine (EPS - IBPS), 75005 Paris, France; 6 Laboratoire de biomathématiques, plateau iB², Faculté de Pharmacie de Paris, Université Paris Descartes, Paris, France; 7 Cellular and Molecular Imaging Facility, INSERM US25, CNRS UMS 3612, Faculté de Pharmacie de Paris, Université Paris Descartes, Université Sorbonne Paris Cité, Paris, France

## Abstract

Brain mural cells form a heterogeneous family which significantly contributes to the maintenance of the blood-brain barrier and regulation of the cerebral blood flow. Current procedures to isolate them cannot specifically separate their distinct subtypes, in particular vascular smooth muscle cells (VSMCs) and mid-capillary pericytes (mcPCs), which differ among others by their expression of smooth muscle actin (SMA). We herein describe an innovative method allowing SMA^+^ VSMCs and SMA^−^ mcPCs to be freshly isolated from the rat cerebral cortex. Using differential RNA-Seq analysis, we then reveal the specific gene expression profile of each subtype. Our results refine the current description of the role of VSMCs in parenchymal cortical arterioles at the molecular level and provide a unique platform to identify the molecular mechanisms underlying the specific functions of mcPCs in the brain vasculature.

## Introduction

As in all vascular beds, brain mural cells are classically defined as the only cells to be entirely embedded in the vascular basement membrane^1^. They are mesenchymal cells that, in the brain, are mostly derived from the neural crests^2,3^. On the basis of their morphology, segmental localization and expression of the smooth muscle actin isoform SMA, several closely related subtypes have been identified in the adult brain, the most abundant ones being the mid-capillary pericytes (mcPCs) and vascular smooth muscle cells (VSMCs)^4–6^. mcPCs are SMA^−^ and can be further subdivided into the morphologically distinct mesh and thin-strand pericytes, while VSMCs are the typical SMA^+^ contractile cells associated with parenchymal arterioles (PAs). Much less abundant subtypes include SMA^+^ ensheathing pericytes, which are uniquely associated with the first downstream vessels branching off PAs (sometimes termed pre-capillary arterioles) and venular pericytes, which display a low SMA expression and are localized only in exiting venules.

Besides their role in vascular development and angiogenesis in the embryonic brain, mural cells make essential contribution to the cerebrovascular physiology in the adult brain^1,7,8^. VSMCs directly participate in the autoregulation of the cerebral blood flow (CBF) and functional hyperaemia^9,10^. Recently, a subset of pericytes, likely corresponding to the contractile SMA^+^ ensheathing pericytes, has also been shown to be pivotal in functional hyperaemia^4,11,12^. By contrast, mcPCs do not significantly contribute to spontaneous vasomotion or CBF regulation, at least by changing the capillary diameter and in physiological conditions^6,13,14^. Instead, they are suspected to be essential for the maintenance of the blood-brain barrier (BBB), clearance of toxic products and regulation of neuroinflammatory processes^8,15–18^. The molecular mechanisms underlying these functions remain however poorly characterized.

A major obstacle in studies of brain mural cells is the difficulty to specifically isolate, identify or genetically target their distinct subtypes since they share the expression of many specific markers such as PDGFRβ, NG2 or RGS5^5,6,19^. Therefore, although they identified several useful markers of brain mural cells such as *Rgs5*, *Abcc9*, *Kcnj8* or *Vtn*, most previous transcriptomic studies^15,20–24^ could not reveal the specific gene expression profiles of distinct subtypes, hampering the characterization of their specific functions. By contrast, one single-cell transcriptomic study has very recently shown that two main subclasses of mural cells, associated respectively with the arterial/arteriolar or capillary/venous segments of the vasculature, can be distinguished in the mouse brain on the basis of their specific gene expression profile^25^.

In the present study, we designed an innovative combination of enzymatic digestion and magnetic-activated cell sorting (MACS) to separately isolate SMA^+^ VSMCs and SMA^−^ mcPCs from the rat brain cortex. Using RNA-Seq, we show that each cell type displays a specific transcriptomic signature and identify genes highly enriched in each cell type as compared to the other. By providing the first gene expression profile of intra-parenchymal VSMCs in the rat brain, our results bring new insight into the molecular mechanisms underlying major functions of cortical PAs such as CBF regulation. This study also identifies numerous genes highly enriched in mcPCs as compared to VSMCs, some of them pointing to specific functions such as cell-cell communications within the neurovascular unit or clearance of toxic products. Our results shed new light upon the heterogeneity among brain mural cells and provide a unique molecular platform, which should be a valuable resource for understanding the specific functions supported by mcPCs and VSMCs in the rodent brain.

## Results

### Arterioles and mid-capillary brain mural cells can be separately isolated from the rat brain

**Figure 1:**
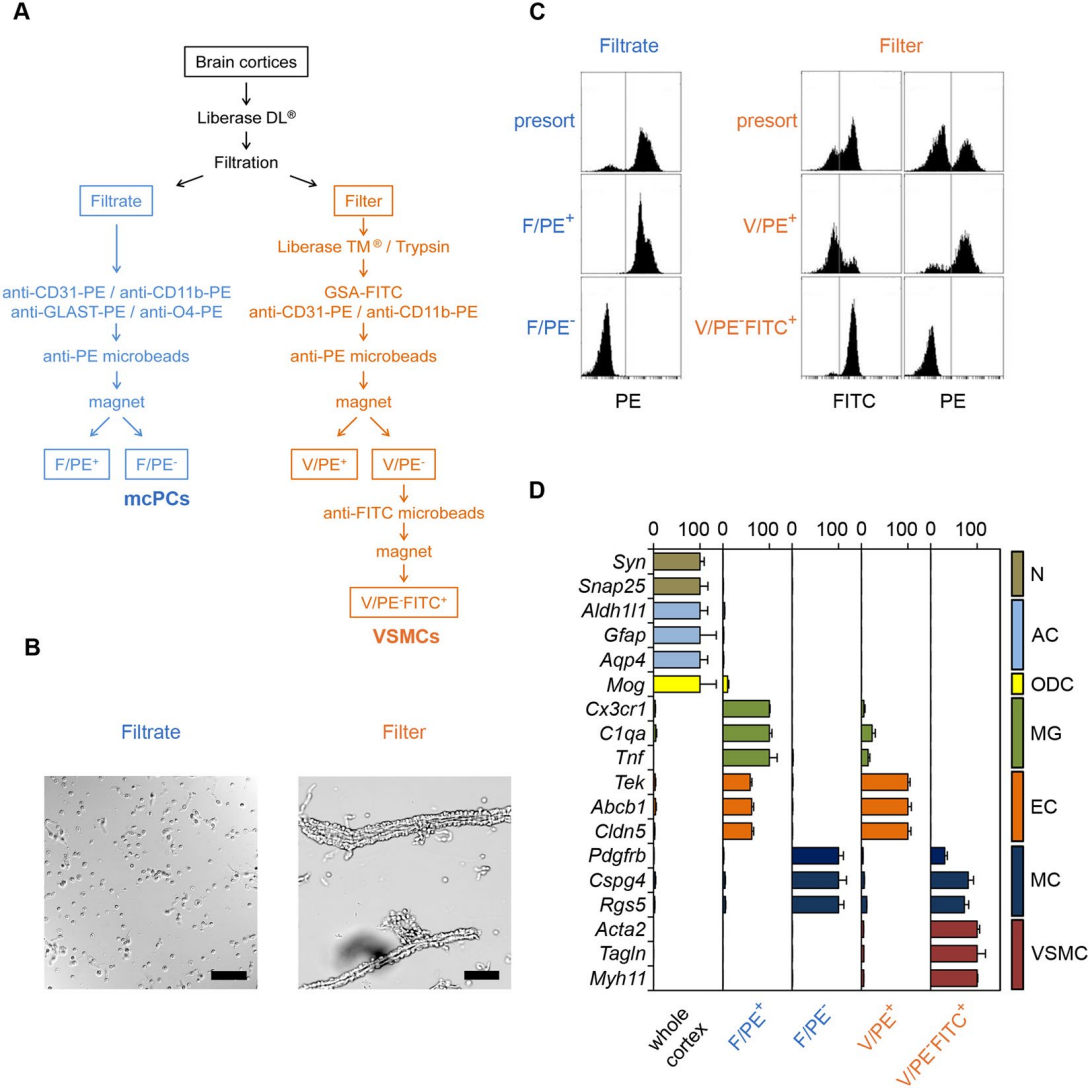
VSMCs and mcPCs can be selectively sorted from the rat cerebral cortex. (**A**) Simplified flowchart of the procedure used to isolate brain mural cells from the mid-capillary bed (Filtrate) and parenchymal arterioles (Filter). mcPCs and VSMCs are recovered in the fractions F/PE^−^ and V/PE^−^FITC^+^, respectively. (**B**) Phase contrast microscopy of the filtrate and arterioles retained on the 10 µm mesh filter (scale bar 50 µm). (**C**) Flow cytometry analysis of magnetically sorted cells. PE fluorescence distribution of magnetically labeled cells from the filtrate before (presort) and after (F/PE^+^, F/PE^−^) magnetic sorting (left panel). FITC and PE fluorescence distributions of magnetically labeled cells from dissociated arterioles before (presort) and after (V/PE^+^, V/PE^-^FITC^+^) magnetic sorting (right panel). (**D**) qRT-PCR analysis confirms the nature and purity of magnetically sorted mural cells. Whole cortex and sorted fractions were analyzed for the relative expression of the indicated specific markers of neurons (N), astrocytes (AC), oligodendrocytes (ODC), microglia (MG), endothelial cells (EC), mural cells (MC) and vascular smooth muscle cells (VSMC). For each gene, values are normalized to the highest value across all samples (mean ± SEM from three independent experiments).

To separately isolate brain mural cells associated with the PAs *vs* mid-capillary bed, we reasoned that PAs should be more resistant to enzymatic dissociation than capillaries, due to their thicker wall and basal lamina. To test this hypothesis, we digested meninges-free brain cortices with the mild enzyme Liberase DL and passed the resulting homogenate on a 10 µm mesh filter. As revealed by phase-contrast microscopy, the material retained on the filter consisted mostly of undigested arterioles (easily identified by their coverage with highly refringent, circumferentially oriented VSMCs) and proximal segments of first downstream vessels. By contrast, the filtrate contained only isolated cells with occasional stretches of 2–3 endothelial cells still attached (Fig. 1B). These results suggested that the enzymatic digestion by Liberase DL was able to completely dissociate the brain parenchyma and mid-capillary bed while leaving brain arterioles mostly intact. Therefore, we designed a cell sorting method to isolate mural cells associated to either vascular segment (see flowchart in Fig. 1A).

The filtrate was expected to contain vascular cells from the mid-capillary bed as well as brain parenchymal cells. Therefore, endothelial cells, microglia/perivascular macrophages, astrocytes and oligodendrocytes were labelled with Phycoerythrin (PE)-conjugated anti-CD31, anti-CD11b, anti-GLAST and anti-O4 antibodies respectively, followed by anti-PE magnetic microbeads and then removed from the filtrate by MACS (Fig. 1A). Neurons were expected to massively die during the procedure, as confirmed by further analysis (see below). Before MACS, 82.4 ± 3.4% of cells were PE^+^ and 17.6 ± 3.4% were PE^−^, as assessed in flow cytometry (Fig. 1C, left panel). Following MACS, the F/PE^+^ fraction was highly enriched in PE^+^ cells while the F/PE^−^ fraction was completely depleted of PE^+^ cells.

The fragments of arterioles were recovered from the filter and completely dissociated using the harsh enzymes Liberase TM and Trypsin. During preliminary experiments, we made the serendipitous observation that the lectin GSA-FITC from *Griffonia simplicifolia*, widely used to specifically label endothelial cells in the mouse brain^26^, did not stain rat brain endothelial cells. Indeed, flow cytometry analysis of cells after double labelling with GSA-FITC and anti-CD31-PE antibody revealed that GSA^+^ and CD31^+^ cells formed two distinct populations (see Supplementary Figure S1). Moreover further analysis of purified GSA-FITC^+^ cells showed that they were VSMCs (see qRT-PCR results below). Therefore, after complete dissociation of the arterioles, endothelial cells, microglia/perivascular macrophages and mural cells were labelled with the anti-CD31-PE antibody, anti-CD11b-PE antibody and lectin GSA-FITC respectively. PE-labelled and FITC-labelled cells were separated sequentially by MACS using anti-PE and anti-FITC magnetic microbeads (Fig. 1A). Before MACS, 57.9 ± 5.5% of cells were FITC^+^, whereas 29.4 ± 3.3% of cells were PE^+^ (Fig. 1C, right panel). Following MACS, the V/PE^+^ fraction was enriched in PE^+^ cells (73.5 ± 2.9%) while the V/PE^−^FITC^+^ fraction was completely depleted from PE^+^ cells and highly enriched in FITC^+^ cells (93.8 ± 0.6%).

### qRT-PCR analysis confirms that isolated cells are respectively VSMCs and mcPCs

The cellular composition of the sorted fractions was assessed by measuring the relative expression of specific cell markers *via* qRT-PCR (Fig. 1D). When compared to the whole cortex, all sorted fractions appeared strongly depleted of neurons, astrocytes and oligodendrocytes. As expected, endothelial and glial cells were collected in the F/PE^+^ and V/PE^+^ fractions while the F/PE^−^ and V/PE^−^FITC^+^ fractions were highly enriched in mural cells, as demonstrated by the expression of the pan-mural markers *Pdgfrb*, *Cspg4* and *Rgs5*. Interestingly, genes encoding contractile proteins including SMA (*Acta2*) were highly expressed in mural cells derived from arterioles (V/PE^−^FITC^+^) but almost undetectable in mural cells derived from the filtrate (F/PE^−^). Thus according to the established localization and SMA expression of mural cells in the rodent brain vasculature, cells from the V/PE^−^FITC^+^ fraction were identified as VSMCs (with a possible minor contribution from SMA^+^ ensheathing pericytes) and cells from the F/PE^−^ fraction were identified as mcPCs.

### VSMCs and mcPCs display specific transcriptomic signatures

The gene expression profile of isolated mcPCs and VSMCs was obtained through RNA-Seq. Normalized counts (in number of reads) and transcripts abundances (in transcripts per million, TPM) were estimated at the gene level and differential gene expression was expressed as Log_2_ of the fold change (LFC) between mcPCs and VSMCs. The whole dataset contained 14,607 genes with at least one read in each sample (after summation over technical replicates). Data were filtered to discard genes with extremely low expression (less than 0.8 TPM in VSMCs, see Methods) yielding a final dataset of 12,203 genes (Supplementary Dataset S1).

**Figure 2:**
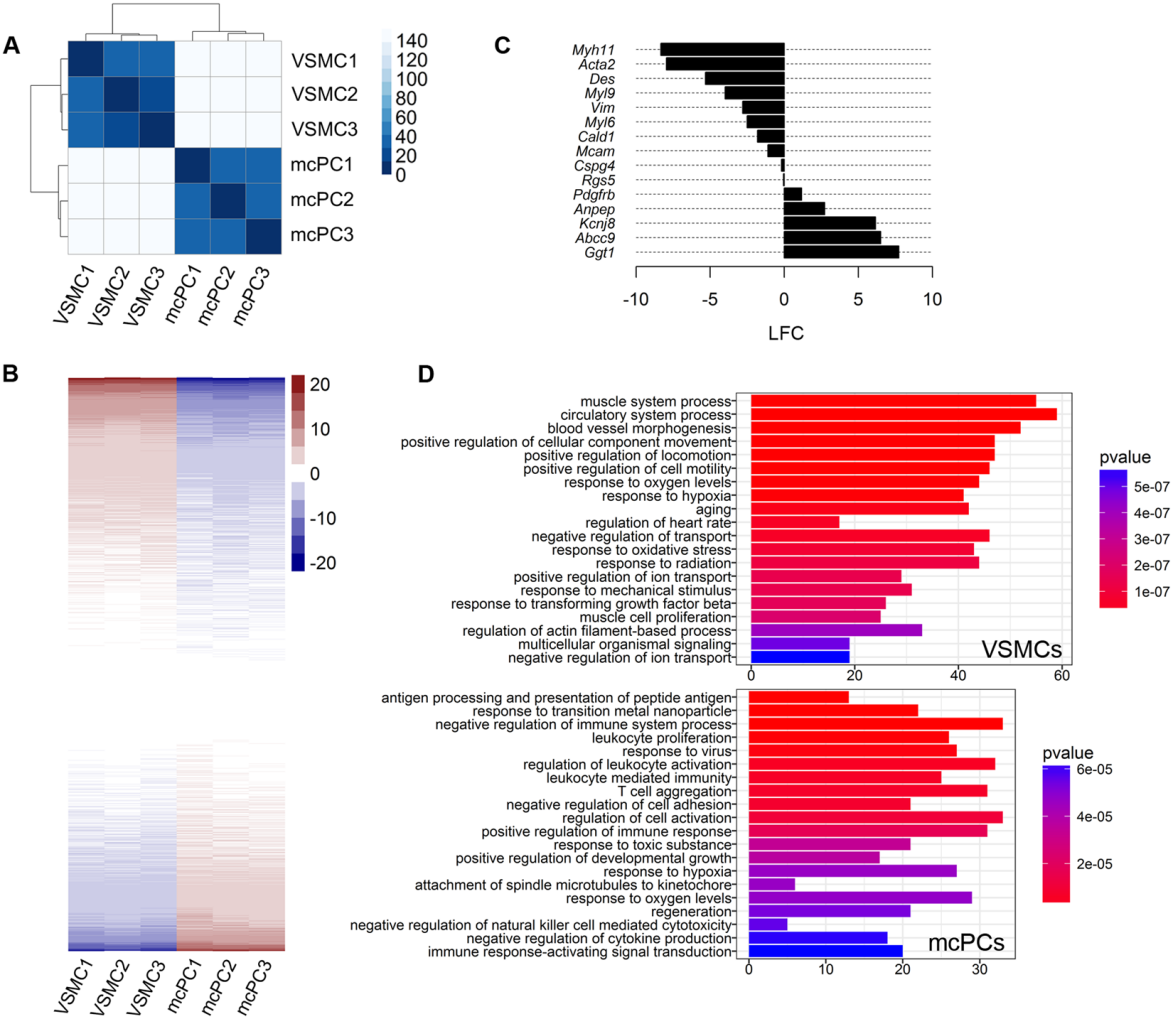
Isolated VSMCs and mcPCs display distinct RNA-Seq transcriptomic signatures. (**A**,**B**) These heatmaps include the 12,203 genes of the final dataset and compare the samples (mcPC1-3 and VSMC1-3) from three independent experiments. (**A**) The heatmap of the sample-to-sample euclidian distances showing high correlation among biological replicates but low correlation between mcPCs and VSMCs. (**B**) The heatmap of standard deviations from mean normalized count values showing that mcPCs and VSMCs display distinct gene expression profiles. (**C**) Differential expression for usual mural markers (LFC is positive for genes enriched in mcPCs, negative for genes enriched in VSMCs). (**D**) Gene Ontology pathways enriched among the genes highly overexpressed in VSMCs (LFC < −2) or mcPCs (LFC > 2). Only the 20 most significantly enriched pathways are shown.

Unsupervised hierarchical clustering of the data showed that mcPCs and VSMCs were clearly distinguishable (Fig. 2A), each one displaying a specific gene expression profile (Fig. 2B). Differential analysis confirmed that 8,264 genes were differentially expressed between mcPCs and VSMCs (adjusted p-values < 0.05), among which 1,897 and 1,552 genes were overexpressed by at least a factor of two in VSMCs (LFC < −1) and mcPCs (LFC > 1), respectively. Usual markers of brain mural cells were found either similarly expressed in VSMCs and mcPCs (such as *Mcam*, *Cspg4*, *Rgs5*, *Pdgfrb*) or selectively enriched in one cell type (Fig. 2C). In particular *Myh11*, *Acta2*, *Des* on the one hand and *Ggt1*, *Abcc9*, *Kcnj8* on the other hand appear to be highly specific for VSMCs and mcPCs respectively. In order to get insight into the specific functions of both cell types, we examined if the most highly overexpressed genes in VSMCs (801 genes with LFC < −2) and mcPCs (631 genes with LFC > 2) were significantly enriched in specific Gene Ontology pathways (Fig. 2D). Pathways related to cell contractility (such as muscle system process, circulatory system process, response to mechanical stimulus or regulation of actin filament-based process), vascular remodelling (such as blood vessel morphogenesis, response to TGFβ or muscle cell proliferation) and response to hypoxia, oxidative stress or aging were highly enriched in VSMCs. By contrast, mcPCs appeared specifically enriched in many pathways related to the regulation of immune processes as well as in pathways involved in the response against virus, metal nanoparticles or toxic substances.

### Fluorescent *in situ* hybridization confirms the differential expression of selected transcripts

**Figure 3:**
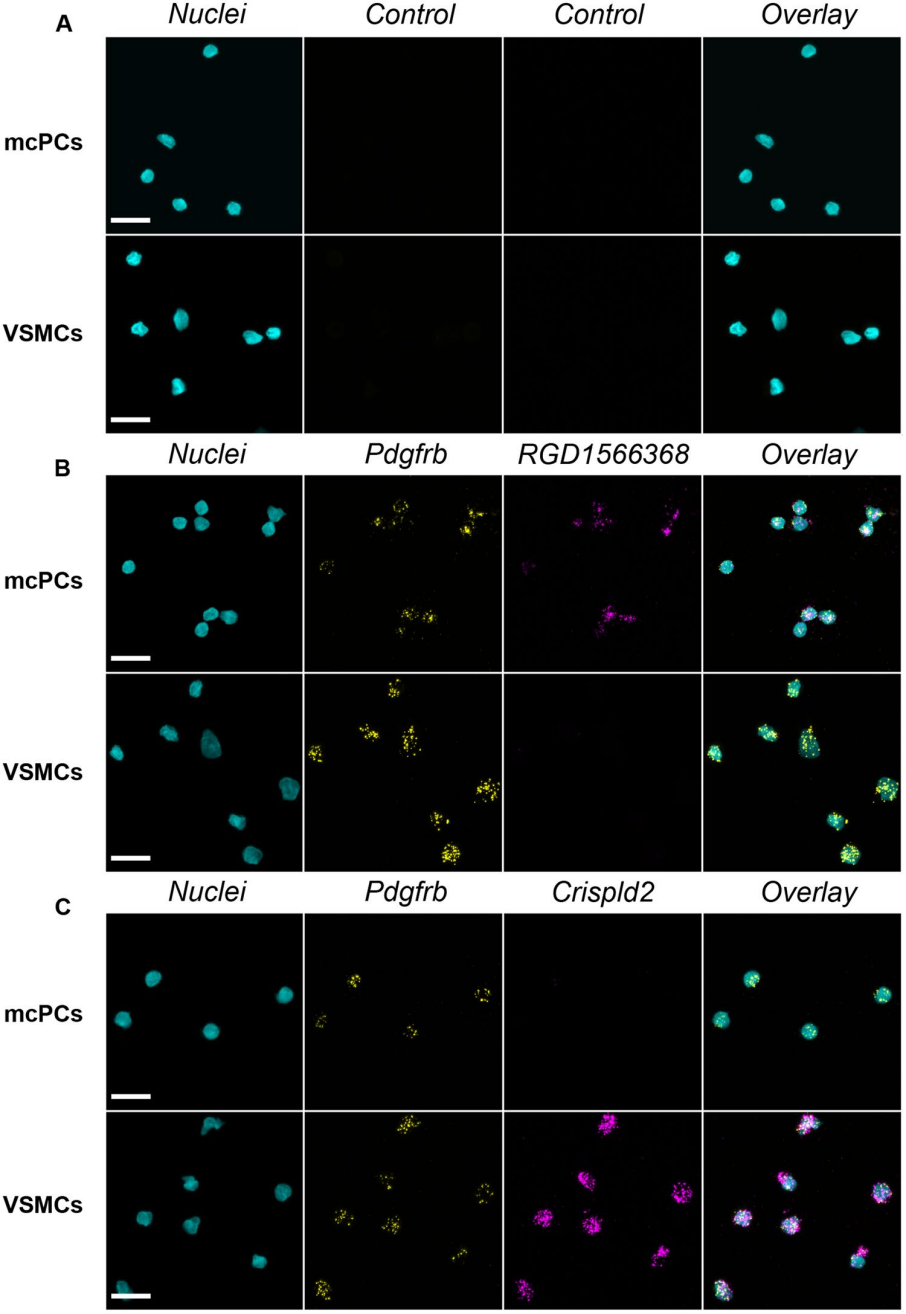
FISH confirms the differential expression of *RGD1566368* and *Crispld2* transcripts in freshly isolated mcPCs and VSMCs. (**A**) A control probe gives virtually no signal in both cell types. (**B**) *Pdgfrb* (yellow)/*RGD1566368* (magenta) double FISH. mcPCs and VSMCs are positive for *Pdgfrb* but only mcPCs are positive for *RGD1566368*. (**C**) *Pdgfrb* (yellow)/*Crispld2* (magenta) double FISH. mcPCs and VSMCs are positive for *Pdgfrb* but only VSMCs are positive for *Crispld2*. (**A**–**C**) Hoechst staining (nuclei) in cyan, scale bar 10 µm.

**Figure 4:**
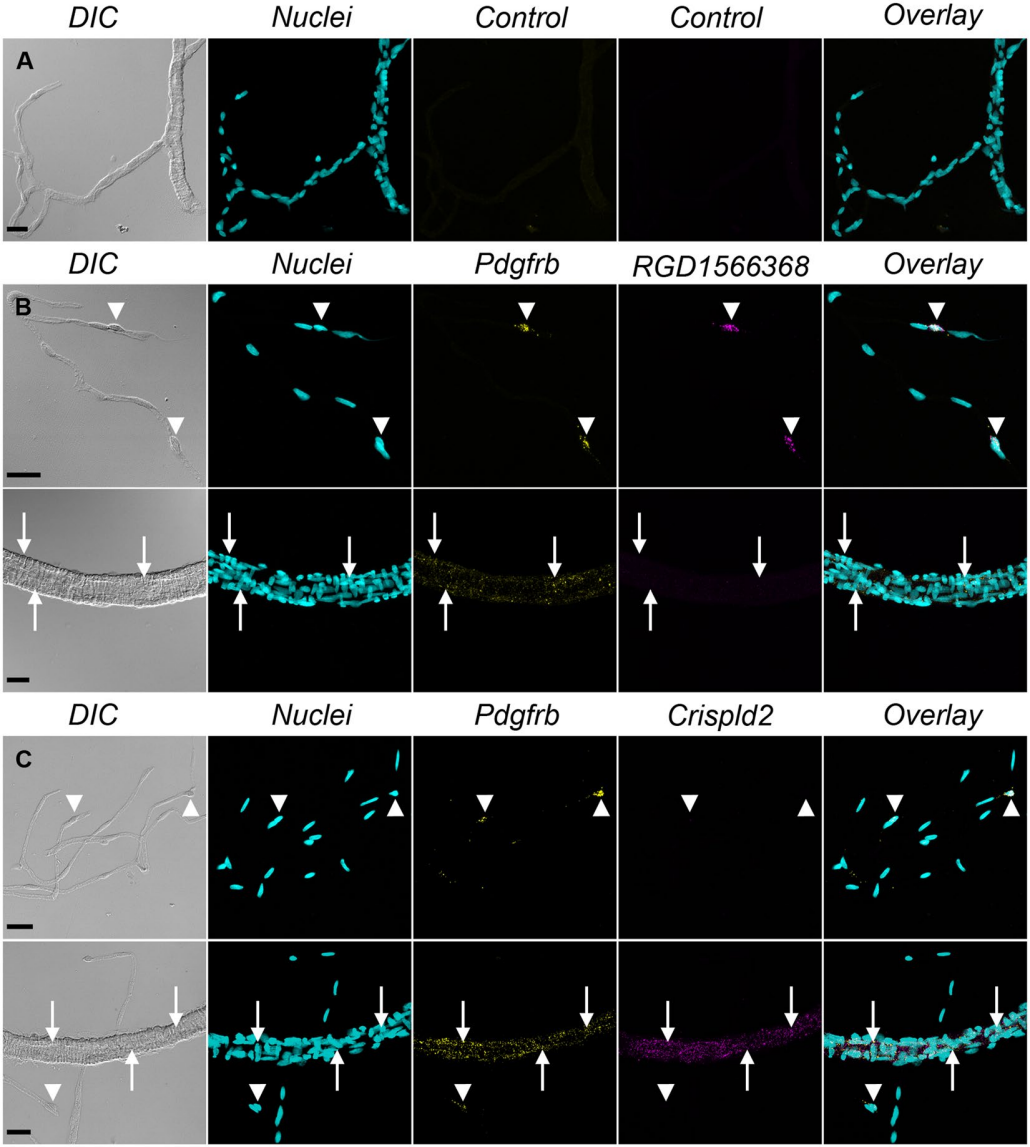
FISH confirms the differential expression of *RGD1566368* and *Crispld2* transcripts in mural cells of mechanically isolated brain vessels. (**A**) A control probe gives virtually no signal. (**B**) *Pdgfrb* (yellow)/*RGD1566368* (magenta) double FISH. Both mcPCs in capillaries (arrowheads) and VSMCs in arterioles (arrow) are positive for *Pdgfrb* but only mcPCs are positive for *RGD1566368*. (**C**) *Pdgfrb* (yellow)/*Crispld2* (magenta) double FISH. Both mcPCs in capillaries (arrowheads) and VSMCs in arterioles (arrow) are positive for *Pdgfrb* but only VSMCs are positive for *Crispld2*. A,B,C, DIC image in gray, Hoechst staining (nuclei) in cyan, scale bar 20 µm.

In order to validate our transcriptomic data, we performed FISH experiments in freshly isolated cells (Fig. 3) and in mechanically isolated whole brain vessels which, contrary to enzymatically isolated vessels, contain a mixture of large vessels and capillaries (Fig. 4). We targeted two transcripts not previously localized in brain mural cells but found to be highly enriched in mcPCs (*RGD1566368*, similar to *Slc6a20*, LFC = 7.31) or VSMCs (*Crispld2*, LFC = −4.93) in the present study. Mural cells were identified by co-labeling of *Pdgfrb* transcripts. A negative control probe gave virtually no staining (Figs 3A and 4A) while the *Pdgfrb* probe strongly labelled mural cells either isolated (Fig. 3B,C) or within mid-capillaries and arterioles (Fig. 4B,C). Moreover, the *RGD1566368* probe strongly stained isolated and *in situ* mcPCs but not isolated or *in situ* VSMCs (Figs 3B and 4B), while the opposite was observed for the *Crispld2* probe (Figs 3C and 4C). Interestingly, in the vascular segment connecting the arterioles to the downstream mid-capillary bed, the expression of *Crispld2* transcripts remained high while *RGD1566368* transcripts were undetectable (see Supplementary Figure S2). These results confirm that our transcriptomic data can be used as a reliable source to identify genes differentially expressed in rat brain mcPCs and VSMCs and that FISH staining in mechanically isolated microvessels is a valuable approach to probe the expression of specific transcripts in different segments of the brain vasculature.

## Discussion

Brain mural cells form a heterogeneous family with several subtypes differing in particular by their morphology and segmental localization as revealed recently by high resolution imaging in fluorescent reporter mouse models^4–6^. How this heterogeneity is reflected at the molecular level remains however largely unexplored. Indeed, many mural markers have been identified^1^ but only for a handful of them has the relative expression among subtypes been examined. PDGFRβ, NG2 and RGS5 are expressed at similar levels in most pericytes and VSMCs, thus forming a pan-mural molecular signature^5,6,19^. By contrast, SMA expression is restricted to VSMCs and ensheathing pericytes^4,5^, while Vitronectin is much more highly expressed in capillary pericytes than in VSMCs^22^.

Several studies provided a whole transcriptome of brain mural cells using microarray or RNA-Seq. Subtractive methods have used a differential transcriptome analysis between whole vessels and endothelial cells^21^ or mural cells deficient vessels^15,20^. However these studies did not select any specific vascular segment so that the obtained transcriptome likely resulted from a mixture of VSMCs and pericytes. More recently, brain mural cells have been freshly isolated using immunocapture of PDGFRβ^+^ cells^24^ or fluorescence-activated cell sorting (FACS) from the brain of *Pdgfrb-eGFP/NG2-DsRed* double reporter mice^22^. Again, both pericytes and VSMCs were expected to contribute to the final dataset since NG2 and PDGFRβ are expressed in both cell types. Altogether these studies have drawn a global molecular landscape of brain mural cells and identified new mural markers but were not designed to unmask the heterogeneity existing among their subtypes. Two studies have directly tackled the issue of mural cells heterogeneity using single-cell transcriptomics^23,25^. One of these studies evidenced two types of mural cells differing by their expression of *Acta2*, but did not analyse their differential gene expression, likely because of its inherent noise^23^. The other one, published during the reviewing process of the present article, provided a deeper comparative analysis of the gene expression profiles of mural cells subtypes and is discussed in more details below^25^.

In the present study, we demonstrated that a mild enzymatic digestion of the rat brain cortex could completely dissociate the mid-capillary bed while leaving intra-parenchymal arterioles mostly intact. This allowed us to separately isolate the mural cells associated with these two vascular segments and to differentially analyse their gene expression profiles. Importantly, our approach does not require the use of transgenic animals expressing a fluorescent reporter^22^ or specific antibodies to label mural cells^24^. Both cell populations expressed pan-mural markers such as *Pdgfrb*, *Cspg4* and *Rgs5* and were highly pure with respect to neurons and glial cells as assessed by qRT-PCR and RNA-Seq. *Acta2* and specific VSMC markers such as *Myh11*, *Cnn1* and *Tagln*^27^ were highly overexpressed in arterioles- *vs* mid-capillary-derived mural cells (LFC ≈ −8). Since it is now established that SMA is highly expressed in VSMCs but undetectable in mcPCs of the rodent brain^4–6^, we concluded that we successfully isolated these two subtypes of brain mural cells. Importantly, although purified cells might also include venular and ensheathing pericytes, these contributions are likely limited since these subtypes appear to be much less abundant than VSMCs and mcPCs in the brain vasculature^4,6^.

Our approach suffers from two main limitations. Firstly, although it brings substantial resolution into the molecular profile of brain mural cells’ family, it cannot separate the two subtypes of mcPCs, namely the mesh and thin-strand pericytes. Of note, even single-cell transcriptomics studies^23,25^ have been unable to distinguish these subtypes, possibly because mesh pericytes are only a tiny fraction of mcPCs. Secondly, mcPCs were slightly contaminated by erythrocytes and endothelial cells. This is a common limitation of cell sorting experiments, in particular during cerebral mural cells’ isolation^22^. However, in the present study, positively sorted VSMCs were much less contaminated than negatively sorted mcPCs, thus offering a simple way to identify and filter out the contaminating transcripts from the dataset (see “RNA-Seq data analysis” in Methods). For future studies a step of red blood cell lysis or an anti-Ly76 antibody (cat.# 130-109-619, Miltenyi) could be used to deplete erythrocytes from the filtrate. Moreover a threshold based on the expression level of the most highly expressed genes in endothelial cells (e.g. *Cldn5*) should be used to obtain the specific transcriptome of mcPCs.

The results from five previous transcriptomic studies of brain mural cells have been recently compiled^22^. Since the overlap between the five datasets was rather limited (with only 3 genes in common), the authors provided a restricted catalogue of 260 genes found in at least two independent studies. Although these data were obtained in the mouse, they can be valuably compared to the present results given the phylogenetic proximity between mice and rats. Interestingly, our results show that, among the 232 genes of this list which were also found in the present study, 10 were highly specific for mcPCs (LFC > 4) such as *Atp13a5*, *Ptn* or *P2ry14*, while 29 were highly specific for VSMCs such as *Susd5*, *Pdlim3*, *Casq2* or the established VSMC markers *Myh11* and *Tagln*. Therefore the present report brings significant resolution into the molecular profile of the mural cells’ family.

Hierarchical clustering following single-cell RNA-Seq has recently identified two distinct subclasses of mural cells in the brain vasculature, namely arterial VSMCs (occurring in a continuum with downstream arteriolar VSMCs) and mid-capillary PCs (occurring in a continuum with downstream venular VSMCs)^25^. Interestingly, among the genes most highly differentially expressed between mural cell types, 85% (62 out of 73) of the genes found enriched in arterial VSMCs and 50% (23 out of 46) of the genes found enriched in PCs in the above-cited study were also found to be enriched respectively in VSMCs (LFC < −1) or mcPCs (LFC > 1) in the present study. These figures were respectively 56% and 43% within the subset of transporters encoding genes. Therefore, although both studies were performed in a different species (rat *vs* mouse) and using a different method (bulk *vs* single-cell RNA-Seq), they reveal a rather similar differential gene expression profile between the two main subclasses of mural cells in the rodent brain. Furthermore, it was shown in the above study that arteriolar VSMCs formed a continuum with upstream arterial VSMCs but abruptly transition to downstream PCs at the arteriole-capillary boundary. This is in agreement with our observation that the expression of the VSMC-specific gene *Crispld2* extends into the lower order arterioles but abruptly vanishes in the mid-capillary bed, while the expression of the mcPC-specific gene *RGD1566368* has the opposite pattern. Although it remains to be confirmed for other VSMC-specific genes, this observation, as long as the fact that no sub-clusters could be evidenced among brain PCs^25^, suggest that the mural cells associated to the arteriole-capillary transition segment (including the so-called ensheathing pericytes) are molecularly closer to VMSCs than to PCs.

Being uniquely embedded in cerebral parenchyma and entirely covered by astrocytic endfeet, PAs likely display a specific phenotype, respond to distinct input signals and eventually support specific functions, as compared to other arteriolar beds^28^. For example they exert a more stringent auto-regulatory response to increased vascular pressure^28–30^ and are uniquely involved in the adaptation of CBF to neuronal activity or functional hyperaemia^9,10^. Yet, information about the molecular repertoire of VSMCs within PAs is currently limited and mainly derived from peripheral or pial arterioles^31–33^. By analysing intracerebral VSMCs isolated from cortices that were carefully cleared of meninges, the present study offers the opportunity to refine our understanding of the molecular mechanisms specifically involved in the control of CBF at the intra-parenchymal level.

In brain PAs, VSMCs modulate the CBF through a purely myogenic response which can be additionally modulated by neuro-humoral factors such as neurotransmitters and arachidonic acid (AA) derivatives^9,10,30^. In the cortex, vasoactive neurotransmitters can be released by neuronal projections from subcortical areas (NA, noradrenaline; 5-HT, serotonin; Ach, acetylcholine; Glu, glutamate) or locally by GABA interneurons (GABA, γ-aminobutyric acid; Ach; VIP, vasoactive intestinal peptide; NPY, neuropeptide Y; SOM, somatostatin; SP, substance P; CGRP, calcitonin gene-related peptide; NO, nitric oxide)^34^. However whether these mediators act directly upon VSMCs and/or induce the local release of vasoactive messengers is still debated^9,34^. Our results show that the expression of the genes encoding receptors for Ach, NA, 5-HT, SOM, SP and Glu in the VSMCs was very low or undetectable, except for *Grik5* which encodes a subunit of kainate receptors (GluK5) unable to form functional homomeric receptors. By contrast *Npy1r*, *Gabbr1* and to a lesser extent *Calcrl* were highly expressed. These results suggest that VSMCs can respond to NPY, GABA and CGRP released from cortical interneurons while vasoactive messengers from subcortical areas need to be relayed locally by neuronal, glial or endothelial cells. Moreover VSMCs were highly enriched in *Avpr1a* and *Crhr2*, suggesting that arginine-vasopressin and urocortin could directly act onto VSMCs in cortical PAs^35^ or, alternatively, that their receptors could act as ligand-independent stretch sensors in the myogenic response^36^.

In VSMCs, NO is a strongly relaxing messenger whose classical mechanism of action involves the stimulation of cGMP synthesis and activation of cGMP-dependent kinases (cGKs)^37^. Interestingly in the present study, VSMCs were found to express guanylate cyclase subunits (*Gucy1a2*, *Gucy1a3*, *Gucy1b3*) but not cGKs (*Prkg1*, *Prkg2*) encoding genes, suggesting that the vasodilatory action of NO in brain PAs either relies on a cGK-independent action of cGMP^38^ or is cGMP-independent, as recently suggested^39^.

Among the genes encoding prostanoid receptors, *Ptgir* was by far the most highly expressed, and *Tbxa2r* was moderately expressed while the expression of *Ptgfr*, *Ptger1* and *Ptger4* was much lower. This suggests that VSMCs in cortical PAs can respond to prostacyclin and thromboxane A2 but that prostaglandin E2 might not exert significant effect directly on them, in line with a recent functional study^12^. The AA derivative 20-HETE is a strong vasoconstrictive messenger, thought to be produced in VSMCs by CYP4A ω-hydroxylases from astrocyte-released AA^40,41^. Since CYP4A encoding transcripts were absent from our transcriptome, we suggest that other cytochromes, such as CYP4F5 and CYP4F6, might be involved in 20-HETE synthesis in brain PAs^42^ or, alternatively, that astrocytes, rather than VSMCs, might be the source of 20-HETE^43^.

Besides refining our understanding of known functions of VSMCs within the brain cortex, the present study also reveals numerous genes currently uncharacterized in these cells. These include for example several recently identified target genes of Notch3 signaling such as *Grip2*, *Xirp1* or *Susd5*^44^. Moreover we identify many genes encoding transcription factors (*Wtip*, *Nrip2*, *Zfp36*, *Fhl5*), secreted proteins (*Crim1*, *Adamtsl1*), intracellular proteins (*Rasd1*, *Tesc*) or membrane receptors (*Olr63*, *Ccrl2*, *Gprc5a*, *Ntrk3*) that were both highly expressed and highly enriched in VSMCs as compared to mcPCs and whose function is currently unknown in cerebral VSMCs.

Studies in transgenic mice with partially disrupted PDGFRβ signalling have suggested that pericytes contribute to the maintenance of the BBB in adulthood and during aging, in particular by promoting a BBB-specific endothelial phenotype characterized by low permeability and immune quiescence^15,16^. Pericytes are also suspected to contribute to brain homeostasis through the regulation of neuro-inflammatory processes^17^ and clearance of toxic products such as the β-amyloid peptide^18,45^. Yet, by contrast with their role during developmental angiogenesis which was analysed in details^1,7,8^, the molecular mechanisms supporting the functions of brain pericytes *in vivo* remain to be characterized in the adult.

In the present study, the gene expression profile of mcPCs was found highly enriched in hundreds of genes with potentially relevant functions. For example *Spon2*, *Col7a1* and *Nid2* encode structural proteins of the extracellular matrix, suggesting that mcPCs participate in vascular homeostasis through unique contributions to the basal lamina^46–48^. Transcripts encoding diverse secreted proteins and membrane receptors, including *Ntn1*, *Unc5b*, *Ptn* and *P2ry14*, were highly enriched in mcPCs. *Ntn1* encodes a secreted protein which can signal onto endothelial cells to promote BBB integrity^49^ and *Unc5b* encodes a known receptor of Netrin-1, suggesting paracrine and autocrine functions of Netrin-1 in mcPCs. Pleiotrophin (*Ptn*) is a multifunctional heparin-binding growth factor with important implication in cancer and angiogenesis^50^, in particular through direct binding to VEGF^51^. Interestingly, we have recently shown that transcripts of *Ptprz1*, encoding the PTN receptor RPTPβ/ζ^50^, are selectively enriched and translated in astrocyte perivascular endfeet^52^, suggesting a functional PTN-RPTPβ/ζ axis between pericytes and astrocytes. *P2ry14* encodes an atypical purinergic receptor which binds specifically UDP and UDP-sugars^53^. The observation that astrocytoma cells can release UDP-glucose in a regulated manner^54^ suggests that astrocytes and pericytes might communicate through this largely unexplored pathway. The gene expression profile of mcPCs was also highly enriched in several genes encoding scavenging or endocytosis receptors such as *Ager*, *Colec12* or *Mrc2*, supporting the suspected role of brain pericytes in the clearance of toxic products^8^. Finally, our pathway analysis support the hypothesis that mcPCs are involved in the regulation of immune processes within the brain^17^.

In conclusion, using an innovative cell sorting strategy, we herein provide the first specific gene expression profile of mcPCs and VSMCs in the rat brain. This molecular database helps to refine our understanding of their specific physiological functions and opens new perspectives to elucidate their contribution to brain homeostasis within the cerebrovascular system.

## Methods

### Animals

Thirty six male 4–5 weeks-old Sprague-Dawley rats were used. They were kept on a 12:12-hour dark:light cycle with *ad libitum* access to food and water. All experiments complied with the ethical rules of the European directive (2010/63/EU) for experimentation with laboratory animals and were approved by the ethics review committee of Paris Descartes University (approval no. 12-187).

### Chemicals

DMEM (cat. # 21885-025), HBSS (cat. # 14175-053), HEPES (cat. # 15630-056) and 0.25% Trypsin-EDTA (cat. # 25200-072) were from Gibco. Fetal Bovine Serum (FBS) (cat. # SV30160.03) was from GE Healthcare Life Science. Liberase DL (cat. # 05401160001), Liberase TM (cat. # 05401119001), Bovine Serum Albumin (BSA) (cat. # A7906), Dextran (cat. # 31390) and DNase I (cat. # DN25) were from Sigma-Aldrich.

### Preparation of brain cell suspensions

Ten rats were used for each isolation (thirty rats in three independent experiments). Deeply anesthetized rats were transcardially perfused with Buffer 1 (HBSS, 10 mmol/L HEPES) at room temperature (RT) for 3 min to remove blood from the brain vasculature. All subsequent steps were done on ice except when indicated. The cortex was dissected, carefully cleared from adhering white matter and meninges and gently crushed in a Petri dish using a glass slide. Tissue pieces from two cortices were pooled, rinsed twice by sedimentation in Buffer 1, then centrifuged (2 min, 600 g) and resuspended in 10 mL of DMEM containing 10 mM HEPES, 0.3 WU/mL Liberase DL and 20 U/mL DNase I. Digestion was performed for 60 min at 37 °C with gentle mechanical trituration with a 10 mL pipette (at 10 and 20 min), then with a P1000 pipet tip (at 30 and 40 min) and finally with a roded glass Pasteur pipette (at 50 and 60 min), to obtain an homogenate with a creamy texture and almost no visible remaining piece. Digestion was stopped by adding 30 mL of DMEM + 10% FBS and the homogenate was centrifuged (5 min, 1,000 g). The supernatant was discarded and the pellet was resuspended in 25 mL of Buffer 1 + 18% BSA and centrifuged (15 min, 2,000 g). The compact myelin disk was eliminated and the pellet resuspended in 50 mL of Buffer 1 + 1% BSA. This suspension was filtered on a 10 µm nylon mesh (cat. # NY1004700, Millipore). Cells from the filtrate were pelleted, resuspended in MACS buffer (PBS, 0.5% BSA, 2 mM EDTA, see below) and stored on ice. Large vessels were recovered in Buffer 1 + 1% BSA from the 10 µm filter, pelleted and digested at RT in 6 mL of Buffer 1 containing 300 µg/mL Liberase TM and 20 U/mL of DNase I for 15 min. The same volume (6 mL) of 0.25% Trypsin/EDTA, 20 U/mL DNase I was added for an additional 30 min. The digestion was stopped by adding 30 mL of DMEM + 10% FBS and undigested vessel fragments were removed by filtration on a 10 µm nylon mesh. Cells were pelleted and resuspended in MACS buffer.

### Magnetic-activated cell sorting

Phycoerythrin (PE) conjugated antibodies, anti-Fluorescein Isothiocyanate (FITC) and anti-PE magnetic microbeads, LS and LD columns were used following the recommendations of the manufacturer (Miltenyi Biotec). The whole procedure was performed at 4 °C and cells were washed in MACS buffer and recovered by centrifugation (10 min, 1,000 g) following each incubation step.

mcPCs were isolated from the filtrate by negative cell sorting. Cells were incubated first in 1.5 mL of MACS Buffer + anti-CD31-PE, anti-CD11b-PE, anti-GLAST-PE and anti-O4-PE antibodies (10% v/v each) for 30 min, then in 500 µL of MACS Buffer + anti-PE microbeads (10% v/v) for 15 min. Cells were then applied to a LS column to retain the F/PE^+^ fraction while the unretained cells were passed on a LD column to remove any remaining labelled cells and further increase the purity of the F/PE^−^ fraction containing mcPCs.

VSMCs were isolated from the large vessels fraction by positive cell sorting. Cells were incubated sequentially in 500 µL of MACS buffer plus 5 µL of the fluorescent lectin conjugate GSA-FITC (cat. # L2895, Sigma Aldrich) for 15 min, in 200 µL of MACS buffer + anti-CD31-PE and anti-CD11b-PE antibodies (10% v/v each) for 15 min and in 80 µL of MACS buffer plus 20 µL of anti-PE microbeads for 15 min. Cells were then passed over a LS column to retain the V/PE^+^ fraction while the unretained cells (fraction V/PE^−^) were incubated for 15 min in 90 µL of MACS buffer plus 10 µL of anti-FITC microbeads and passed over a LS column. VSMCs were recovered in the retained fraction (V/PE^−^FITC^+^).

### Flow cytometry

Cells were stained with 7-AAD (4 mg/L for 20 min on ice) and analyzed on a BD Accuri™ C6 (BD Biosciences) flow cytometer. Debris and dead cells were excluded from the analysis by gating events on FSC/SSC and 7-AAD fluorescence respectively.

### qRT-PCR

Following MACS, cells were immediately lysed and total RNA was extracted using the RNeasy® Micro Kit (Qiagen). Reverse transcription was performed on 100 ng of total RNA using random primers (cat # N8080127) and the Superscript II Reverse Transcriptase (cat # 18064022) following the supplier’s recommendations (Life Technologies). For qPCR, 8 µL of cDNA diluted 1/20 was mixed with 10 µL of SYBR Green fluorescence detection solution (Thermo Fisher Scientific) and 1 µL of each primer (Eurogentec) (see Supplementary Table S1 for primers’ sequence). qRT-PCR was performed in an ABI Prism® 7900HT Sequence Detection System (Applied Biosystems) using the following program: 2 min at 50 °C, 10 min at 95 °C, 40 cycles of amplification (15 sec at 95 °C, 45 sec at 60 °C). All primers had amplification efficiency close to 100% as checked by the analysis of standard dilution curves (slope close to −3.33) so that the relative expression of a gene *X* to the housekeeping gene *Tbp* could be calculated by 2^−ΔCt^ where ΔCt = Ct(*X*) − Ct(*Tbp*) and Ct is the threshold cycle value.

### RNA-Seq

Library preparation and Illumina sequencing were performed at the École normale supérieure genomic core facility (IBENS, Paris, France). Messenger (polyA^+^) RNAs from mcPCs and VSMCs obtained in three independent experiments (biological replicates) were purified from 100 ng of total RNA using oligo(dT). All samples had RNA integrity numbers ranging from 6.40 to 8.50 as assessed by Agilent Bioanalyzer (Agilent Technologies). Libraries were prepared using the strand specific RNA-Seq library preparation TruSeq Stranded mRNA kit (Illumina). Libraries were multiplexed by six on a run and each library was sequenced four times (technical replicates). A 75 bp single-end read sequencing was performed on NextSeq 500 device (Illumina). A mean of 65 ± 15 million reads passing Illumina quality filter was obtained for each of the six samples.

### RNA-Seq data analysis

Before mapping, poly N read tails were trimmed, reads ≤ 40 bases were removed, and reads with quality mean ≤ 30 were discarded using the Eoulsan pipeline^55^. Transcript abundances (in Transcripts Per Million, TPM) and counts were estimated using Salmon (version 0.8.0)^56^ and the Ensembl annotation file Rattus_norvegicus.Rnor_6.0.cdna.all.fa.gz (available at ftp://ftp.ensembl.org/pub/release-87/fasta/rattus_norvegicus/cdna/). Results were summed within genes using the Tximport package (version 1.2.0)^57^ and abundances and counts at the gene level were used for further analysis. The DESeq. 2 package (version 1.14.1)^58^ was used for normalizing counts and estimating differential gene expression which was expressed as LFC, defined as the Log_2_ of the fold change between mcPCs and VSMCs, with corresponding adjusted p-values. The whole dataset contained 14,607 genes with at least one read in each sample (after summation over technical replicates).

The expression of selected transcripts, known to be highly and selectively expressed in neurons, glial cells and endothelial cells of the rodent brain^24^, ranged from undetectable to 0.64 TPM in VSMCs (see Supplementary Table S3). Similarly low values were obtained in mcPCs except for the endothelial-specific transcripts which were more abundant in mcPCs than in VSMCs. We therefore filtered the data by removing all transcripts whose mean abundance was lower than an empirical threshold of 0.8 TPM in VSMCs (2,389 genes). Moreover, the 15 transcripts the most highly enriched in mcPCs (LFC > 7.8) were mainly from erythrocyte-specific genes, such as globin subunits encoding genes, and were also discarded yielding a final dataset of 12,203 genes (Supplementary Dataset S1).

### Mechanical isolation of brain microvessels

Two rats were used for each isolation (six rats in three independent experiments). The rats were anesthetized and transcardially perfused with Buffer 1 as described above. Cortices were cleared of meninges and gently crushed between two glass slides. Tissue pieces were recovered in 30 mL of Buffer 1 and triturated in a Potter-Thomas homogenizer using 10 strokes at 400 rpm. The homogenate was centrifuged, washed once in Buffer 1 and resuspended in Buffer 1 + 17.5% Dextran. After centrifugation at 2,500 g for 15 min, myelin was eliminated and the pellet resuspended in Buffer 1 + 1% BSA. This suspension was filtered on a 10 µm mesh filter and vessels’ fragments were recovered from the filter in Buffer 1 + 1% BSA.

### Fluorescent *in situ* RNA hybridization (FISH)

Double FISH was performed using the RNAscope® multiplex fluorescent assay (Advanced Cell Diagnostics) with C1 and C2 probes following the manufacturer’s recommendations. Briefly, freshly isolated mcPCs and VSMCs or mechanically isolated brain microvessels were adhered onto glass slides previously coated with Cell-Tac (Corning), fixed in 4% paraformaldehyde (15 min at 4 °C) and dehydrated in an ascending series of ethanol. After treatment in Pretreat solution 3 (isolated cells) or 4 (microvessels) for 15 min at RT, slides were hybridized with FISH probes for 2 h at 40 °C and labelling was revealed using FITC- and Cy3-labeled amplifiers for C1 and C2 probes respectively. Slides were eventually mounted in Fluoromount containing DAPI for nuclei counterstaining. Z-stacks with 0.7 µm steps were acquired on a Leica TCS-SP8 confocal microscope (Leica Microsystems) equipped with a x40 objective (numerical aperture = 1.30), using constant acquisition parameters for each probe. Images shown are maximum intensity projections. Hybridization of a probe targeting Bacillus subtilis dihydrodipicolinate reductase (dapB) transcripts was used as negative control. The references of the probes used are given in Supplementary Table S2.

## Electronic supplementary material

Supplementary information

Supplementary dataset S1

## Data Availability

Raw and processed RNA-Seq data are available in the NCBI Gene Expression Omnibus (http://www.ncbi.nlm.nih.gov/geo/) under the accession number GSE100355.

## References

[1] Armulik A, Genové G, Betsholtz C (2011). Pericytes: developmental, physiological, and pathological perspectives, problems, and promises. Dev. Cell.

[2] Etchevers HC, Vincent C, Le Douarin NM, Couly GF (2001). The cephalic neural crest provides pericytes and smooth muscle cells to all blood vessels of the face and forebrain. Dev. Camb. Engl..

[3] Korn J, Christ B, Kurz H (2002). Neuroectodermal origin of brain pericytes and vascular smooth muscle cells. J. Comp. Neurol..

[4] Grant, R. I. *et al*. Organizational hierarchy and structural diversity of microvascular pericytes in adult mouse cortex. *J*. *Cereb*. *Blood Flow Metab*. *Off*. *J*. *Int*. *Soc*. *Cereb*. *Blood Flow Metab*. 271678X17732229, 10.1177/0271678X17732229 (2017). 10.1177/0271678X17732229 PMC6399730 28933255

[5] Hartmann DA (2015). Pericyte structure and distribution in the cerebral cortex revealed by high-resolution imaging of transgenic mice. Neurophotonics.

[6] Hill RA (2015). Regional Blood Flow in the Normal and Ischemic Brain Is Controlled by Arteriolar Smooth Muscle Cell Contractility and Not by Capillary Pericytes. Neuron.

[7] Gaengel K, Genové G, Armulik A, Betsholtz C (2009). Endothelial-mural cell signaling in vascular development and angiogenesis. Arterioscler. Thromb. Vasc. Biol..

[8] Sweeney MD, Ayyadurai S, Zlokovic BV (2016). Pericytes of the neurovascular unit: key functions and signaling pathways. Nat. Neurosci..

[9] Attwell D (2010). Glial and neuronal control of brain blood flow. Nature.

[10] Iadecola C, Nedergaard M (2007). Glial regulation of the cerebral microvasculature. Nat. Neurosci..

[11] Hall CN (2014). Capillary pericytes regulate cerebral blood flow in health and disease. Nature.

[12] Mishra A (2016). Astrocytes mediate neurovascular signaling to capillary pericytes but not to arterioles. Nat. Neurosci..

[13] Damisah EC, Hill RA, Tong L, Murray KN, Grutzendler J (2017). A fluoro-Nissl dye identifies pericytes as distinct vascular mural cells during *in vivo* brain imaging. Nat. Neurosci..

[14] Fernández-Klett F, Priller J (2015). Diverse functions of pericytes in cerebral blood flow regulation and ischemia. J. Cereb. Blood Flow Metab. Off. J. Int. Soc. Cereb. Blood Flow Metab..

[15] Armulik A (2010). Pericytes regulate the blood-brain barrier. Nature.

[16] Bell RD (2010). Pericytes control key neurovascular functions and neuronal phenotype in the adult brain and during brain aging. Neuron.

[17] Rustenhoven J, Jansson D, Smyth LC, Dragunow M (2017). Brain Pericytes As Mediators of Neuroinflammation. Trends Pharmacol. Sci..

[18] Sagare AP (2013). Pericyte loss influences Alzheimer-like neurodegeneration in mice. Nat. Commun..

[19] Bondjers C (2003). Transcription profiling of platelet-derived growth factor-B-deficient mouse embryos identifies RGS5 as a novel marker for pericytes and vascular smooth muscle cells. Am. J. Pathol..

[20] Bondjers C (2006). Microarray analysis of blood microvessels from PDGF-B and PDGF-Rbeta mutant mice identifies novel markers for brain pericytes. FASEB J. Off. Publ. Fed. Am. Soc. Exp. Biol..

[21] Daneman R (2010). The mouse blood-brain barrier transcriptome: a new resource for understanding the development and function of brain endothelial cells. PloS One.

[22] He L (2016). Analysis of the brain mural cell transcriptome. Sci. Rep..

[23] Zeisel A (2015). Brain structure. Cell types in the mouse cortex and hippocampus revealed by single-cell RNA-seq. Science.

[24] Zhang Y (2014). An RNA-sequencing transcriptome and splicing database of glia, neurons, and vascular cells of the cerebral cortex. J. Neurosci. Off. J. Soc. Neurosci..

[25] Vanlandewijck M (2018). A molecular atlas of cell types and zonation in the brain vasculature. Nature.

[26] Wälchli T (2015). Quantitative assessment of angiogenesis, perfused blood vessels and endothelial tip cells in the postnatal mouse brain. Nat. Protoc..

[27] Owens GK, Kumar MS, Wamhoff BR (2004). Molecular regulation of vascular smooth muscle cell differentiation in development and disease. Physiol. Rev..

[28] Cipolla MJ (2014). Increased pressure-induced tone in rat parenchymal arterioles vs. middle cerebral arteries: role of ion channels and calcium sensitivity. J. Appl. Physiol. Bethesda Md 1985.

[29] Cipolla MJ, Smith J, Kohlmeyer MM, Godfrey JA (2009). SKCa and IKCa Channels, myogenic tone, and vasodilator responses in middle cerebral arteries and parenchymal arterioles: effect of ischemia and reperfusion. Stroke.

[30] Kim KJ (2015). Astrocyte contributions to flow/pressure-evoked parenchymal arteriole vasoconstriction. J. Neurosci. Off. J. Soc. Neurosci..

[31] Davis MJ, Hill MA (1999). Signaling mechanisms underlying the vascular myogenic response. Physiol. Rev..

[32] Hill MA, Meininger GA (2012). Arteriolar vascular smooth muscle cells: mechanotransducers in a complex environment. Int. J. Biochem. Cell Biol..

[33] Walsh MP, Cole WC (2013). The role of actin filament dynamics in the myogenic response of cerebral resistance arteries. J. Cereb. Blood Flow Metab. Off. J. Int. Soc. Cereb. Blood Flow Metab..

[34] Hamel E (2006). Perivascular nerves and the regulation of cerebrovascular tone. J. Appl. Physiol. Bethesda Md 1985.

[35] Du W, Stern JE, Filosa JA (2015). Neuronal-derived nitric oxide and somatodendritically released vasopressin regulate neurovascular coupling in the rat hypothalamic supraoptic nucleus. J. Neurosci. Off. J. Soc. Neurosci..

[36] Storch U, Schnitzler MMY, Gudermann T (2012). G protein-mediated stretch reception. Am. J. Physiol. - Heart Circ. Physiol..

[37] Lincoln TM, Dey N, Sellak H (2001). Invited review: cGMP-dependent protein kinase signaling mechanisms in smooth muscle: from the regulation of tone to gene expression. J. Appl. Physiol. Bethesda Md 1985.

[38] Francis SH, Busch JL, Corbin JD (2010). cGMP-Dependent Protein Kinases and cGMP Phosphodiesterases in Nitric Oxide and cGMP Action. Pharmacol. Rev..

[39] Stobart JLL, Lu L, Anderson HDI, Mori H, Anderson CM (2013). Astrocyte-induced cortical vasodilation is mediated by D-serine and endothelial nitric oxide synthase. Proc. Natl. Acad. Sci. USA.

[40] Mulligan SJ, MacVicar BA (2004). Calcium transients in astrocyte endfeet cause cerebrovascular constrictions. Nature.

[41] Berg RMG (2016). Myogenic and metabolic feedback in cerebral autoregulation: Putative involvement of arachidonic acid-dependent pathways. Med. Hypotheses.

[42] Roman RJ (2002). P-450 metabolites of arachidonic acid in the control of cardiovascular function. Physiol. Rev..

[43] Gebremedhin D (2016). Expression of CYP 4A ω-hydroxylase and formation of 20-hydroxyeicosatetreanoic acid (20-HETE) in cultured rat brain astrocytes. Prostaglandins Other Lipid Mediat..

[44] Fouillade C (2013). Transcriptome analysis for Notch3 target genes identifies Grip2 as a novel regulator of myogenic response in the cerebrovasculature. Arterioscler. Thromb. Vasc. Biol..

[45] Winkler EA, Sagare AP, Zlokovic BV (2014). The pericyte: a forgotten cell type with important implications for Alzheimer’s disease?. Brain Pathol. Zurich Switz..

[46] Martins, V. L. *et al*. Suppression of TGFβ and Angiogenesis by Type VII Collagen in Cutaneous SCC. *J*. *Natl*. *Cancer Inst*. **108** (2016). 10.1093/jnci/djv293 26476432

[47] Yokota K (2017). Periostin Promotes Scar Formation through the Interaction between Pericytes and Infiltrating Monocytes/Macrophages after Spinal Cord Injury. Am. J. Pathol..

[48] Zhu L-H (2015). Mindin regulates vascular smooth muscle cell phenotype and prevents neointima formation. Clin. Sci. Lond. Engl. 1979.

[49] Podjaski C (2015). Netrin 1 regulates blood-brain barrier function and neuroinflammation. Brain J. Neurol..

[50] Papadimitriou E (2016). Pleiotrophin and its receptor protein tyrosine phosphatase beta/zeta as regulators of angiogenesis and cancer. Biochim. Biophys. Acta.

[51] Héroult M (2004). Heparin affin regulatory peptide binds to vascular endothelial growth factor (VEGF) and inhibits VEGF-induced angiogenesis. Oncogene.

[52] Boulay A-C (2017). Translation in astrocyte distal processes sets molecular heterogeneity at the gliovascular interface. Cell Discov..

[53] Lazarowski ER, Harden TK (2015). UDP-Sugars as Extracellular Signaling Molecules: Cellular and Physiologic Consequences of P2Y14 Receptor Activation. Mol. Pharmacol..

[54] Kreda SM, Seminario-Vidal L, Heusden Cvan, Lazarowski ER (2008). Thrombin-promoted release of UDP-glucose from human astrocytoma cells. Br. J. Pharmacol..

[55] Jourdren L, Bernard M, Dillies M-A, Le Crom S (2012). Eoulsan: a cloud computing-based framework facilitating high throughput sequencing analyses. Bioinforma. Oxf. Engl..

[56] Patro R, Duggal G, Love MI, Irizarry RA, Kingsford C (2017). Salmon provides fast and bias-aware quantification of transcript expression. Nat. Methods.

[57] Soneson C, Love MI, Robinson MD (2015). Differential analyses for RNA-seq: transcript-level estimates improve gene-level inferences. F1000Research.

[58] Love MI, Huber W, Anders S (2014). Moderated estimation of fold change and dispersion for RNA-seq data with DESeq. 2. Genome Biol..

