# Supplementary material for: Isolation and differential transcriptome of vascular smooth muscle cells and mid-capillary pericytes from the rat brain
Source: Sci Rep. 2018 Aug 16;8:12272. doi: 10.1038/s41598-018-30739-5 (PMC6095852; doi:10.1038/s41598-018-30739-5)
Supplement: Supplementary file 1 — Supplementary information [file 41598_2018_30739_MOESM1_ESM.pdf]

Supplementary Information

Isolation and differential transcriptome of vascular smooth muscle cells and mid-capillary pericytes from the rat brain.

Stéphanie Chasseigneaux, Yasmine Moraca, Véronique Cochois-Guégan, Anne-Cécile Boulay, Alice Gilbert, Stéphane Le Crom, Corinne Blugeon, Cyril Firmo, Salvatore Cisternino, Jean-Louis Laplanche, Emmanuel Curis, Xavier Declèves, Bruno Saubaméa.

Table S1. Sequences of forward (F) and reverse (R) primers used in qRT-PCR experiments.

Gene	Primer sequence
Syn	*F* : GCCACGGACCCAGAGAACATTA<br>*R* : TTCAGGAAGCCAAACACCACT
Snap25	*F* : CTGCCCGTGTGGTGGATGAA<br>*R* : ATGCCGCTCACCTGCTCTAG
Aldh1l1	*F* : GTGTGAGGTGCTTCCAGATGAC<br>*R* : GCTGTACCCTCTGCGATCAG
Gfap	*F* : AGGCAGAAGCTCCAAGATGAAAC<br>*R* : TCTCCAGATCCACACGAGCCAA
Aqp4	*F* : TGAATCCAGCTCGATCCTTTGG<br>*R* : TCCACGTCAGGACAGAAGACAT
Mog	*F* : AGACCACTCCTACCAAGAAGAAG<br>*R* : ATAGGCACAAGGGCAATGAGAG
Cx3cr1	*F* : TCGCCCCAGTTCATGTTCAC<br>*R* : GGACGAAGCCCAGGATGTT
C1qa	*F* : GAGCACCCAACGGGAAGGA<br>*R* : GTCTCCTTTAAGACCTCGGATACC
Tnf	*F* : CGGTCCCAACAAGGAGGAGA<br>*R* : AGAAGATGATCTGAGTGTGAGGGT
Tek	*F* : GGGGGAGTGGAGTGAAGAACT<br>*R* : CCGTCCACGATTGTCCAAGAAAC
Abcb1a	*F* : CAACCAGCATTCTCCATAATA<br>*R* : CCCAAGGATCAGGAACAATA
Cldn5	*F :* CAGCCCCCAACCCACAGC<br>*R :* CACCCAGCCTACCAGACACA
Pdgfrb	*F* : GGGCAATGATGTGGTCAACTT<br>*R* : GGGCACTCCGAAGAGGTAAT
Cspg4	*F* : ACCAGCTAGAGGTAGTCCAGG<br>*R* : GACACCATCACCAAGTAGCCAG
Rgs5	*F* : GCCACACTCATGCCTGGAAA<br>*R* : GGCTTCTCCGGCTTCTCATTA
Acta2	*F* : ACACGGCATCATCACCAACT<br>*R* : CGCCTCCGTTAGCAAGGT
Tagln	*F* : CCAGACTGTTGACCTCTTTGAA<br>*R* : CTCCACGGTAGTGTCCATC
Myh11	*F* : ATCGTGAAGACCAGTCCATTCT<br>*R* : GATGCCACCACAGCCAAATACT
Tbp	*F* : TGCACAGGAGCCAAGAGTGAA<br>*R* : CACATCACAGCTCCCCACCA

Table S2. References of probes used in FISH experiments.

Accession number	Gene Name	ACD Probe name	Target Region	Channel
NM_031525.1	*Pdgfrb*	Rn- Pdgfrb	999-1993	C2
NM_138518.2	*Crispld2*	Rn-Crispld2	602-1535	C1
ENSRNOT00000007467	*RGD1566368*	Rn-Slc6a20-O2	219-1347	C1 or C2

Table S3. Abundances (summarized at the gene level and expressed in Transcripts Per Million, TPM) of selected contaminating transcripts from unwanted cells in the whole data set. Abundances of selected transcripts specific for mural cells are indicated in bold characters for matter of comparison.

	Ensembl_ID	Gene name	TPM in VSMCs	TPM in mcPCs
Astrocyte markers	ENSRNOG00000016043	*Aqp4*	0.05	0.09
	ENSRNOG00000005697	*Slc6a11*	n.d.	n.d.
	ENSRNOG00000032871	*Mlc1*	n.d.	n.d.
Neuron markers	ENSRNOG00000001837	*Sst*	n.d.	n.d.
	ENSRNOG00000036802	*Snhg11*	0.14	0.10
	ENSRNOG00000009768	*Npy*	n.d.	n.d.
Oligodendrocyte markers	ENSRNOG00000028648	*Olig1*	n.d.	n.d.
	ENSRNOG00000000775	*Mog*	0.50	0.31
	ENSRNOG00000018700	*Mobp*	0.33	0.38
Microglial markers	ENSRNOG00000055156	*Tnf*	n.d.	n.d.
	ENSRNOG00000011205	*Ccl3*	n.d.	n.d.
	ENSRNOG00000011406	*Ccl4*	n.d.	n.d.
Endothelial markers	ENSRNOG00000045811	*Cldn5*	0.64	15.44
	ENSRNOG00000020173	*Tie1*	0.55	2.76
	ENSRNOG00000008587	*Tek*	0.14	1.39
**Mural markers**	**ENSRNOG00000018461**	***Pdgfrb***	**614.46**	**388.24**
	**ENSRNOG00000017208**	***Cspg4***	**387.89**	**95.21**
	**ENSRNOG00000002730**	***Rgs5***	**6298.45**	**1703.21**

Dataset S1

Dataset S1.xlsx is available as a separate file in the supplemental material.

Final dataset of the RNA-Seq experiment (n = three independent experiments). Each row corresponds to one gene and columns are entitled as follows:

- Ensembl_Gene_ID (Ensembl ID of the gene)
- Entrez_Gene_ID (Entrez Gene ID of the gene)
- Gene_Symbol (gene official name)
- Gene_Description (gene full name)
- MeanTPM_VSMC (mean abundance in VSMCs in Transcripts Per Million)
- MeanTPM_mcPC (mean abundance in mcPCs in Transcripts Per Million)
- MeanCounts_VSMC (mean counts in VSMCs in number of reads)
- MeanCounts_mcPC (mean counts in mcPCs in number of reads)
- MeanLength_VSMC (mean transcript length in VSMCs)
- MeanLength_mcPC (mean transcript length in mcPCs)
- baseMean (mean normalized counts in VSMCs and mcPCs)
- MeanCountsNorm_VSMC (mean normalized counts in VSMCs)
- MeanCountsNorm_mcPC (mean normalized counts in mcPCs)
- log2FoldChange (log2 of the expression Fold Change between mcPCs and VSMCs)
- LFC SE (standard error of the log2 fold change)
- LFC pvalue (p-value associated to log2 fold change)
- LFC padj (Benjamini-Hochberg adjusted p-value associated to log2 fold change)
- GO Location (cell compartment of the encoded protein as given by Gene Ontology)
- GO Family (family of the encoded protein as given by Gene Ontology)

Figure S1

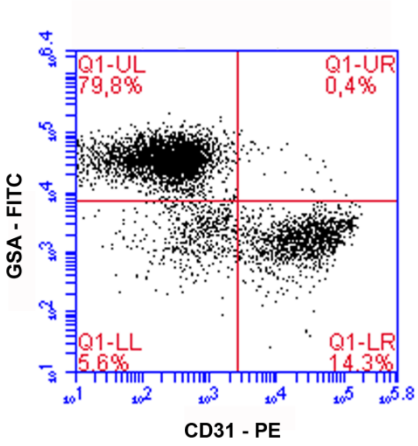

Figure S1
Flow cytometry analysis of cells dissociated from cerebral arterioles and doubly labelled with the fluorescent lectin conjugate GSA-FITC and the endothelial cell specific anti-CD31-PE antibody. Cells are either CD31^{+}GSA^{-}, CD31^{-}GSA^{+}, or CD31^{-}GSA^{-} with virtually no CD31^{+}GSA^{+} cells. Therefore, contrary to what is known in the mouse brain, the lectin GSA-FITC does not stain endothelial cells in the rat brain. Moreover, qRT-PCR analysis reveals that GSA-FITC^{+} cells are VSMCs (see Results).

Figure S2

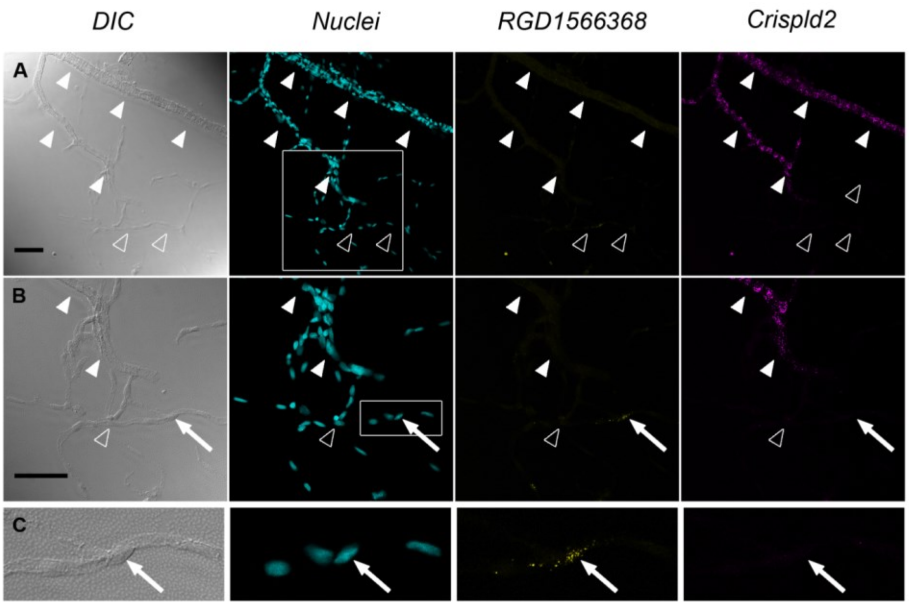

Figure S2

Double FISH staining of *RGD1566368* (yellow) and *Crispld2* (magenta) transcripts in mural cells of mechanically isolated brain vessels. **A**, Image of a continuous vascular fragment extending from an arteriole and its first downstream segment (arrowheads) to the mid-capillary bed (empty arrowheads). **B**, Enlargment of the boxed area in A. **C**, Enlargment of the boxed area in B. The expression of *Crispld2* is high in the proximal arteriolar segment (arrowheads) but vanishes in the distal capillary segment (empty arrowheads) where a *RGD1566368* expressing pericyte is visible (arrow). No mural cells coexpressing *Crispld2* and *RGD1566368* are found in the arteriolar-capillary transition. **A**,**B**,**C**, DIC image in gray, Hoechst staining (nuclei) in cyan, scale bar 50 µm.
